# The BAX/BAK-like protein BOK is a prognostic marker in colorectal cancer

**DOI:** 10.1038/s41419-017-0140-2

**Published:** 2018-01-26

**Authors:** Steven Carberry, Beatrice D’Orsi, Naser Monsefi, Manuela Salvucci, Orna Bacon, Joanna Fay, Markus Rehm, Deborah McNamara, Elaine W. Kay, Jochen H. M. Prehn

**Affiliations:** 1 Department of Physiology and Medical Physics, Centre for Systems Medicine, Royal College of Surgeons in Ireland, Dublin 2, Ireland; 2 Department of Biomedical Sciences, University of Padova, Padova, Italy; 3 Department of Surgery, Beaumont Hospital, Dublin 9, Ireland; 4 Department of Pathology, Beaumont Hospital and Royal College of Surgeons in Ireland, Dublin 9, Ireland; 5 Institute of Cell Biology and Immunology, University of Stuttgart, Allmandring 31, 70569 Stuttgart, Germany

## Abstract

The intrinsic or mitochondrial apoptosis pathway is controlled by the interaction of antiapoptotic and pro-apoptotic members of the BCL-2 protein family. Activation of this death pathway plays a crucial role in cancer progression and chemotherapy responses. The BCL-2-related ovarian killer (BOK) possesses three BCL-2 homology domains and has been proposed to act in a similar pro-apoptotic pathway as the pro-apoptotic proteins BAX and BAK. In this study, we showed that stage II and III colorectal cancer patients possessed decreased levels of BOK protein in their tumours compared to matched normal tissue. BOK protein levels in tumours were also prognostic of clinical outcome but increased BOK protein levels surprisingly associated with earlier disease recurrence and reduced overall survival. We found no significant association of BOK protein tumour levels with ER stress markers GRP78 or GRP94 or with cleaved caspase-3. In contrast, BOK protein levels correlated with Calreticulin. These data indicate BOK as a prognostic marker in colorectal cancer and suggest that different activities of BOK may contribute to cancer progression and prognosis.

## Introduction

Colorectal cancer (CRC) is a leading cause of cancer-related mortality. Current treatment options for patients are dependent on disease stage at diagnosis and consist of surgery and adjuvant or palliative chemotherapy. However, drug resistance, both innate and acquired, remains an obstacle in the effective treatment of this disease. The B-cell lymphoma gene 2 (BCL-2) proteins, consisting of both pro-apoptotic and antiapoptotic members, play a crucial role in carcinogenesis and responses to chemotherapy by controlling the activation of the mitochondrial or intrinsic apoptosis pathway^1^. The pro-apoptotic multidomain proteins BCL-2-associated protein x (BAX) and BCL-2-antagonist/killer (BAK) are activated during apoptosis and form pores in the mitochondrial outer membranes that allow for the release of pro-apoptotic factors^2–4^. This process is commonly referred to as mitochondrial outer membrane permeabilisation (MOMP). BAX and BAK have been explored in CRC regarding their potential as prognostic biomarkers^5^. Genetic mutations in the *Bak* gene are rare in CRC^6^, and reports whether BAX expression levels are associated with improved or poor survival in CRC patients are contradictory^5,7,8^. Their poor prognostic potential in CRC may be due to the fact that BAX and BAK functions are largely redundant, as double *bax/bak* deletion is required to prevent apoptosis in most cell types^9^, whereas single gene deletions have minimal effects on cell survival^10^. Previous studies have also shown that BAX and BAK protein levels are generally exceeding the levels of antiapoptotic BCL-2 proteins in colon cancer cells and CRC patient tumour samples, suggesting that single inhibition of BAX or BAK may not be sufficient to induce resistance in colon cancer cells and that rather the complex biology of the BCL-2 interaction network determines cell survival^11,12^.

The BCL-2-related ovarian killer (BOK) possesses, similar to BAX and BAK, three BCL-2 homology (BH1–3) domains, and thus it has been proposed to act in a similar pro-apoptotic pathway. However, the role of BOK in cancer cell death is still controversial and its role as a prognostic biomarker in CRC has not yet been explored. BOK overexpression results in MOMP, caspase-3 activation, nuclear fragmentation and apoptosis in several cell systems^13–19^. Recently, BOK has been attributed pro-apoptotic properties during defects in endoplasmic reticulum (ER)-associated degradation (ERAD), where it promotes MOMP^20,21^. ERAD is upregulated in response to ER stress resulting in unfolded proteins being retro-translocated from the ER lumen to the cytosol for their ubiquitylation and degradation, thereby contributing in resolving ER stress. Other groups, including ours, demonstrated that BOK is dispensable for most forms of apoptotic cell death in mouse cortical neurons, haematopoietic and mouse embryonic fibroblasts^22–25^ or even exerts pro-survival effects during Ca^2+^-mediated neuronal injury and ER stress^21,22,24,26^. Hence, the function of BOK in apoptosis signalling is still a matter of debate and may be stimulus- and tissue-specific^27^. Physiologically, BOK regulates ER Ca^2+^ homeostasis and has been shown to bind to the IP_3_ receptors at the ER, protecting them from proteolytic cleavage^28^. In addition, BOK may also regulate cell proliferation^29^.

So far, only one study provided evidence for a potential role of BOK as a tumour suppressor in cancer, demonstrating that the *Bok* gene was silenced in many human cancers^30^. Owing to its unexplored role in CRC, we here investigated BOK expression in CRC and subsequently explored whether it was associated with clinical outcome and examined whether BOK protein levels correlated with ER stress.

## Results

### *Bok* mRNA levels are not prognostic of overall survival in CRC

**Fig. 1:**
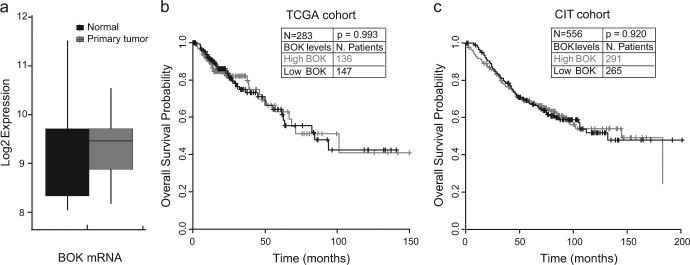
*Bok* gene expression is not prognostic of overall survival in CRC **a** *Bok* gene expression levels compared in *n* = 26 matched tumour and normal tissues from the TCGA COAD cohort. There is no significant difference between normal and tumour expression levels (Student’s *t*-test). **b** OS analysis in the TCGA patient cohort. *n* = 283 patients from the TCGA cohort were divided into high and low *Bok*-expressing groups based on the average *Bok* expression level. Survival analysis showed no significant correlation between *Bok* gene expression and OS (log-rank test). **c** OS analysis in the CIT patient cohort. In this cohort, *n* = 556 patients were divided into high and low *Bok*-expressing groups based on the average *Bok* expression level. Survival analysis of the two groups also showed no significant correlation between *Bok* gene expression and OS (log-rank test)

We first investigated whether *Bok* mRNA levels were altered in CRC by analysing the *Bok* gene expression levels in 26 matched normal and primary tumour samples in the The Cancer Genome Atlas (TCGA) Colon Adenocarcinoma (COAD) cohort. Student’s *t*-test was carried out and revealed no statistical difference in *Bok* mRNA expression between normal and tumour tissues (Fig. 1a). Next we explored *Bok* gene expression in relation to overall survival (OS) in two different sets of CRC patients: 283 and 556 patients derived from the TCGA COAD and Cartes d’Identité des Tumeurs (CIT) cohorts, respectively. Survival analysis of these two groups showed no significant correlation between *Bok* gene expression and OS (Fig. 1b, c). Similar findings were observed when correlating *Bok* gene expression with disease-free survival (DFS) (data not shown, *p* = 0.079049 for the CIT cohort; log-rank test).

### *Bok* gene methylation sites are hypomethylated in both normal and tumour tissues but are not prognostic of overall survival in CRC

**Fig. 2:**
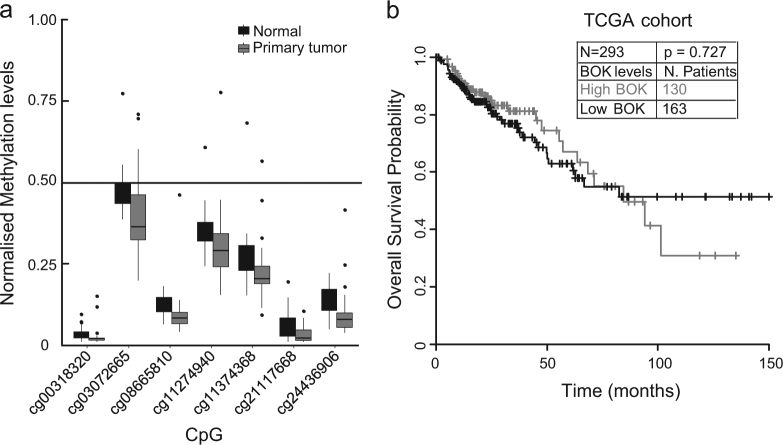
Survival analysis in relation to Bok gene methylation **a** Methylation analysis was carried out on 38 matched normal and primary tumour samples from the TCGA COAD cohort. Seven of the 14 *Bok* methylation sites were significantly different in tumour vs normal tissue (Student’s *t*-test). 0.5 was used as cutoff (line) to group methylation levels, with values >0.5 representing hypermethylation and values <0.5 representing hypomethylation. Only the seven *Bok* methylation sites significantly different between tumour vs normal tissues are shown. Note that all differentially expressed *Bok* methylation sites were hypomethylated in both tumour and normal tissues. **b** Survival analysis in relation to *Bok* gene methylation sites. The values of the differentially methylated *Bok* gene methylation site were averaged and used for survival analysis. Log-rank test result showed no significant difference in the relation to OS in *n* = 293 patients of the TCGA cohort

In order to investigate whether *Bok* gene methylation sites were altered between normal vs tumour tissue, we carried out methylation analysis on 38 matched normal and primary tumour samples from the TCGA COAD cohort. Gene methylation analysis demonstrated that 7 of the 14 *Bok* methylation sites were significantly different between normal and tumour samples in the TCGA COAD cohort and that those 7 sites were all hypomethylated in both tumour and normal tissues (Fig. 2a; Student’s *t*-test). We next explored whether *Bok* gene methylation sites correlated with OS. Methylation values of the seven identified sites were averaged, and the mean of the averages was used as cutoff to assign high or low levels of methylation. Survival analysis revealed that *Bok* methylation status did also not correlate with patient OS (Fig. 2b). Similar findings were observed when investigating individual methylation sites (data not shown).

### CRC patient samples show decreased expression levels of BOK

**Fig. 3:**
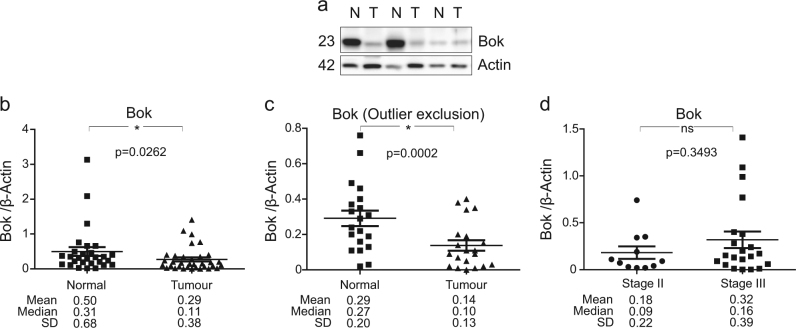
Levels of Bok protein in CRC patient cohort **a** Representative western blotting comparing the levels of BOK in colorectal tumour (T) and matched normal (N) colonic tissue samples. β-Actin was used as a loading control. **b** Scatter plot depicting the protein levels of BOK in colorectal tumour and matched normal tissue. Differences between tumour and matched normal (*n* = 28) tissues were assessed by Wilcoxon signed-rank test. The mean, median and standard deviation (SD) were stated below the panels. *p ≤ 0.05 compared to normal tissue. **c** Scatter plot of the BOK protein levels following outlier identification analysis (for details, see Materials and methods section) in colorectal tumour and matched normal tissue. Differences between tumour and matched normal (*n* = 20) tissue were assessed by Wilcoxon signed-rank test. The mean, median and standard deviation (SD) were stated below the panels. *p ≤ 0.05 compared to normal tissue. **d** Scatter plot of the BOK protein levels in Stage II (*n* = 11) vs Stage III (*n* = 22) CRC patient tissue. Differences between levels of BOK protein in Stage II and Stage III CRC samples were assessed by Mann–Whitney *U* test. The mean, median and SD are stated below the panels

The pro-apoptotic activity of BOK has recently been reported to be controlled post-translationally. BOK stability is highly dependent on BOK binding to IP_3_ receptors and is furthermore controlled by components of the ER stress and ERAD pathway through which it is ubiquitinated and degraded by the proteasome^21,25,26^. Thus we next evaluated the expression levels of BOK protein and the key ER stress markers 78 kDa glucose-regulated protein/binding immunoglobulin protein (GRP78), heat shock protein 90 kDa beta member 1 (GRP94) and Calreticulin in fresh-frozen tumour resections of CRC patients. Because no BOK antibodies are available to detect BOK protein by immunohistochemistry, we employed quantitative western blotting to determine whether BOK protein levels varied in tumour and matched normal tissue (*n* = 28 matched sample) of resected and quality-assured tumour cases of Stages II and III CRC patients (Table 1). We found that BOK protein levels were significantly reduced in tumour samples compared to their matched normal tissues (Fig. 3a, b; *p* = 0.0262, two-sided Wilcoxon signed-rank test). Because the data suggested a high degree of heterogeneity in BOK protein levels between patients, we also subjected the data to a structured unbiased approach for potential outlier identification (for details, see Materials and methods section). Using this approach, we identified eight potential outliers. Despite the exclusion of these potential outliers from the analysis, BOK protein levels still resulted in significantly decreased tumour compared to matched normal tissues (Fig. 3c; *p* = 0.0002, two-sided Wilcoxon signed-rank test).

**Table 1:** Details of the disease stage, chemotherapy treatment, sex and disease outcome of the 33 patients within the study

Patient	Sex	Location	Chemo treatment	RCPath	Staging	Outcome
1	F	Right	None	B	II	Good
2	F	Right	None	B	II	Good
3	M	Left	None	B	II	Good
4	F	Right colon	Unknown	C	III	Good
5	F	Rectosigmoid	5FU/Leu	C	III	Good
6	M	Caecal	None	C	III	Good
7	M	Sigmoid	5FU/Leu	B	II	Good
8	M	Anterior resection	50.4/28 + 5FU	A	II	Good
9	M	Sigmoid	5FU/Leu	C	III	Good
10	M	Caecal	None	B	II	Good
11	M	Sigmoid	None	B	II	Good
12	F	Caecal	Unknown	C	III	Good
13	F	Rectosigmoid	5FU/Leu	C	III	Good
14	F	Sigmoid	FOLFOX	C	III	Good
15	M	Left/colonic	5FU/Leu	C	III	Good
16	M	Anterior resection	50.4/28 + 5FU	B	II	Good
17	M	Anterior resection	50.4/28 + 5FU	C	III	Good
18	M	Right	None	B	II	Poor
19	M	Caecal	5FU/Leu	C	III	Poor
20	F	Sigmoid	FOLFOX	C	III	Poor
21	F	Rectal	None	B	II	Poor
22	F	Sigmoid	FOLFOX	C	III	Poor
23	M	Sigmoid	5FU/Leu	C	III	Poor
24	M	Right	5FU/Leu	C	III	Poor
25	M	Caecal	FOLFIRI	C	III	Poor
26	M	Sigmoid	None	B	II	Poor
27	M	Rectal	Unknown	C	III	Poor
28	F	Ascending	FOLFOX	C	III	Poor
29	F	Rectal	FOLFOX/ Avastin	C	III	Poor
30	M	Colonic	5FU/Leu/Avastin/Irin/Cetux	C	III	Poor
31	F	Caecal	5FU/folinic acid	C	III	Poor
32	M	Rectal	5FU/Leu	C	III	Poor
33	M	Anterior resection	50.4/30 + 5FU	C	III	Poor

*5FU* 5-Fluorouracil, *Fol* Fluorouracil, *Irin* Irinotecan, *Leu* Leucovorin, *Cetux* Cetuximab, *50.4/28* total amount of 50.4 Gy radiotherapy in 28 fractions, *None* no chemotherapy received, *RCPath* Royal College of Pathologists grade

### Expression of ER stress markers does not correlate with BOK protein levels

**Fig. 4:**
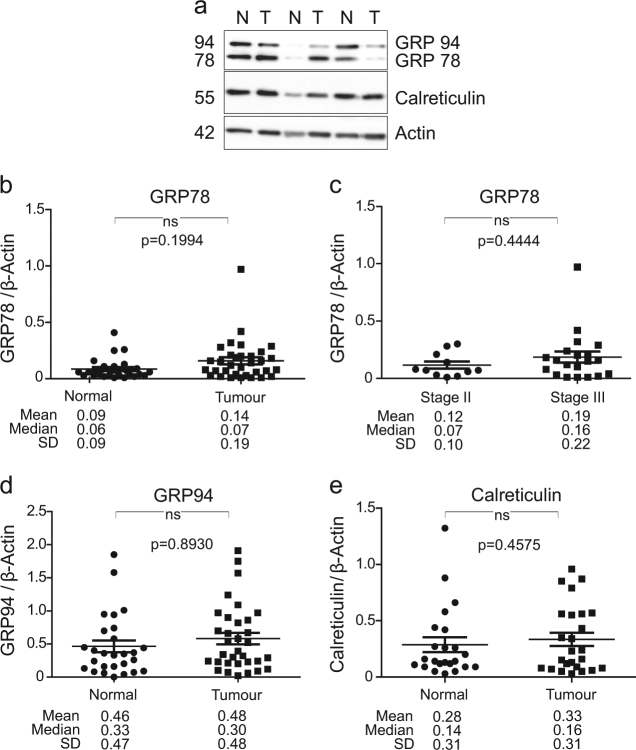
Levels of ER stress proteins in a cohort of CRC patients **a** Representative western blotting comparing the levels of ER stress proteins GRP78, GRP94 and Calreticulin in colorectal tumour (T) and matched normal (N) colonic tissue samples. β-Actin was used as a loading control. Scatter plots depicting the protein levels of GRP78 **b**, GRP94 **d** and Calreticulin **e** in colorectal tumour and matched normal (*n* = 27 for GRP78 and GRP94 and *n* = 24 for Calreticulin) tissues. Differences between tumour and matched normal tissues were assessed by Wilcoxon signed-rank test. The mean, median and standard deviation (SD) are stated below the panels. **c** Scatter plot of the GRP78 protein levels in Stage II (*n* = 11) vs Stage III (*n* = 20) CRC patient tissue. Differences between protein levels of GRP78 in Stage II and Stage III CRC samples were assessed by Mann–Whitney *U* test. The mean, median and SD are stated below the panels

Next, the protein levels of GRP78, GRP94 and Calreticulin were determined in tumour and matched normal tissues from the same cohort of CRC patients. Quantitative western blotting revealed no differential expression of these ER stress markers between tumour and matched normal CRC patient samples (Fig. 4; two-sided Wilcoxon signed-rank test). Further analysis identified a lack of correlation between tumour levels of BOK and ER stress markers, GRP78 and GRP94. However, we observed a positive correlation between tumour levels of BOK and Calreticulin (Table 2; *p* = 0.0072, two-sided Wilcoxon signed-rank test). We also observed no correlation between BOK levels and cleaved caspase-3 levels (Table 2).

**Table 2:** Correlation analysis between BOK protein expression levels and ER stress markers (GRP78, GRP94 and Calreticulin) and indicator of apoptosis pathway (cleaved caspase-3) in CRC

Bok tumour correlation	GRP78	GRP94	Calreticulin	Cleaved Caspase 3
Number of XY pairs	32	32	24	24
Pearson *r*	−0.1516	−0.2383	0.5338	0.0014
*P*-value (two-tailed)	0.4074	0.1890	0.0072	0.8629
*P*-value summary	ns	ns	**	ns

Note that due to exhaustion of tissues reduced matched pairs were available for Calreticulin and cleaved caspase-3

We also examined whether protein levels of BOK and ER stress markers were associated with CRC disease stage. Patient samples were subdivided by tumour stage in Stage II (*n* = 11) and Stage III (*n* = 22) disease. No statistically significant difference was observed in the levels of BOK and GRP78 between Stages II and III disease tumour samples (Figs 3d and 4c; Mann–Whitney *U* test). No statistically significant difference was also observed in the levels of GRP94 and Calreticulin between Stages II and III disease tumour samples (data not shown).

### BOK protein levels are a prognostic marker candidate of clinical outcome in CRC patients

We finally assessed whether protein levels of BOK and/or ER stress markers levels associated with clinical outcome. Samples were divided into two subgroups based on the clinical response of patients: (i) favourable outcome, patients displaying no cancer mortality and/or no disease recurrence within the 4-year follow-up time period; and (ii) unfavourable outcome, patients exhibiting disease recurrence and/or death from disease within the 4-year follow-up time period.

**Fig. 5:**
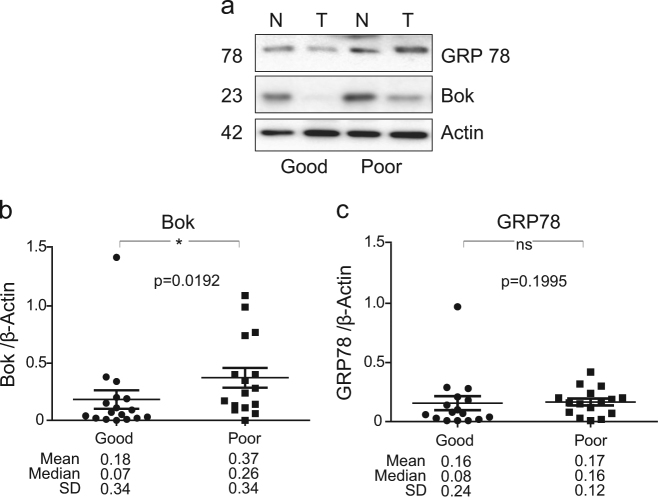
BOK but not GRP78 as a potential prognostic biomarker in stage II/III CRC patients **a** Representative western blotting comparing the levels of BOK and GRP78 in colorectal tumour (T) and matched normal (N) colonic tissue samples of CRC patients with good and poor outcomes. β-Actin was used as loading control. **b**, **c** Scatter plots depicting protein levels of BOK **b** and the ER stress protein GRP78 **c** in the total CRC patient cohort with good (*n* = 17 for BOK, *n* = 16 for GRP78) vs poor (*n* = 16 for BOK, *n* = 16 for GRP78) outcomes. BOK protein levels were significantly increased in those patients who observed a poor clinical outcome compared with patients with good clinical outcome (*p = 0.0192; Mann–Whitney *U* Test). The mean, median and SD are stated below the panels. In contrast, GRP78 did not correlate with clinical outcome (*p* = 0.1995; Mann–Whitney *U* Test)

Interestingly, BOK protein levels were increased in tumour samples from colorectal patients with unfavourable outcome compared to favourable outcome (Fig. 5a, b; Mann–Whitney *U* test, *p* = 0.0192). In contrast, no significant association was observed between GRP78, GRP94 and Calreticulin expression and clinical outcome (Fig. 5a, c and data not shown; Mann–Whitney *U* test). To investigate potential germline effects, we lastly examined whether expression of BOK in normal tissue correlated with clinical outcome. Correlation analysis showed no association between BOK expression levels and disease stage and/or clinical outcome in normal tissue (data not shown).

## Discussion

This study identifies the BAX/BAK-like BCL-2 protein family member BOK as a promising prognostic marker in CRC. We demonstrated that BOK protein levels in colorectal tumour were significantly decreased compared to their matched normal samples, suggesting that reduced BOK protein levels may contribute to carcinogenesis and tumour establishment. More importantly, we also show that elevated BOK levels correlated with recurrence and unfavourable clinical outcome in Stages II and III CRC patients. This apparent discrepancy may reflect the contributions of different activities of BOK during tumour establishment vs recurrence and metastasis.

The BCL-2 proteins, consisting of both pro-apoptotic and antiapoptotic members, play a central role in cancer progression and in responses to both genotoxic and targeted therapies^1^. For this reason, they are frequently studied for their use as prognostic biomarkers^5,12,31^. Of the BAX/BAK-like BCL-2 protein subfamily, the role of BOK is just recently being explored in the context of human cancers. A somatic copy-number alterations study investigating 3131 cancer specimens suggested that BOK may act as a tumour suppressor across several human cancers^30^. Lack of BOK expression was also observed in several multiple myeloma cell lines^21^. In line with these findings, we found that BOK protein levels were significantly lower in tumour tissue of CRC patients compared to matched normal samples, suggestive of a role as a tumour suppressor. However, when correlating BOK protein levels to clinical outcome, we found that increasing BOK protein levels were associated with unfavourable outcome. Interestingly, analysis of two independent large-scale cohorts also indicated that *Bok* mRNA expression was not prognostic in CRC, suggesting that BOK is primarily post-translationally regulated. These findings were supported by an analysis of *Bok* gene methylation sites, which also failed to correlate with clinical outcome.

BOK has been shown to possess pro-apoptotic functions when overexpressed, promoting cytochrome-*c* release, caspase-3 activation and nuclear fragmentation^13–17^. Exact mechanisms of its pro-death functions are controversial; however, a recent study proposed that BOK’s pro-death function is controlled by ER stress and the ERAD pathway^21^. Nevertheless, there is evidence that the function of BOK may indeed have diverted from those of BAX and BAK. As mentioned above, double *Bax/Bak* deletion is required to prevent apoptosis in most cell types^27^, suggesting that BOK is not required for apoptosis in most settings. Indeed, we did not observe a direct correlation of cleaved caspase-3 levels and BOK protein levels in the CRC tumour samples. Unlike BAX and BAK, BOK is predominately found in the membranes of the ER and Golgi apparatus and has been shown to interact with IP_3_ receptors 1 and 2. In fact, its stability is highly dependent on BOK binding to the IP_3_ receptors, and only free BOK is controlled by the ERAD pathway through which it is ubiquitinated and degraded by the proteasome^21,32^. Of note, it has been shown that increased expression of IP_3_ receptors in different types of cancer, including CRC, may be responsible for metastasis formation and tumour aggressiveness and may be employed as biomarkers^33,34^. Furthermore, BOK may also regulate cell proliferation. BOK has been found in proliferating trophoblast cells during early placental development and was localised to the nucleus of proliferating cells and regulated the expression of cyclin E1^29^. It is therefore conceivable that proliferative effects or effects on ER calcium homeostasis rather than pro-apoptotic effects of BOK associate with poor prognosis in CRC. Furthermore, antiapoptotic effects have also been associated with BOK^21,22,24,26^.

Our study also argues against a direct association of ER stress and BOK protein levels in CRC. Levels of the ER-resident chaperone GRP78, which is the primary sensor of ER stress and plays a key role in the unfolded protein response, failed to correlate with BOK protein levels or with clinical outcome. Although we have not specifically investigated the signalling components of the ERAD pathway in relation to BOK protein levels, ERAD is activated in response to several ER stress stimuli^35^. However, the possibility remains that ERAD is activated independent of a broad ER stress response^21^ and, through such mechanisms, regulates BOK protein levels in CRC. In contrast, BOK levels correlated positively with Calreticulin levels. As Calreticulin is not only ER resident but also detectable on the cell surface of cancer cells where it promotes antigen presentation and cellular phagocytic uptake^36^, it possible that this correlation relates to ER stress-independent functions.

In summary, this study highlights the ‘BAX/BAK-like’ protein BOK as a prognostic marker in CRC, with increased BOK tumour levels indicating unfavourable clinical outcome in Stage II/III CRC patients.

## Materials and methods

### Patient cohort

Patient tissue samples were collected and stored in the APOCOLON colorectal tissue biobank at Beaumont Hospital (Dublin, Ireland). Informed consent was received from all patients and ethical approval, for use of the stored material, was granted by Beaumont Hospital Ethics (Medical Research) Committee. Snap-frozen colorectal tumour and matched normal tissue from surgical resections of 33 CRC patients were collected. In 17 cases, patients had a good outcome, which was defined as no mortality or disease recurrence within the 4-year follow-up period, while 16 cases had a poor outcome, specifically, disease recurrence or death from disease within that timeframe. Matched normal tissue was available from *n* = 28 patients. Clinical follow-up was obtained for all patients and patient characteristics are summarised in Table 1.

### Western blotting

For clinical samples, tissue was lysed in ice-cold tissue lysis buffer (50 mmol/L HEPES (pH7.5), 150 mmol/L NaCl, 5 mmol/L Na-EDTA) and protease inhibitor cocktail (Calbiochem, Hampshire, UK) followed by mechanical homogenisation on ice. Following centrifugation (14000 × *g* for 10 min), supernatant was collected and stored at −80 °C until further use.

For the cell line standards, HeLa cell pellets were collected. Cell pellets were then lysed with sodium dodecyl sulfate (SDS) lysis buffer (Protein lysis buffer: 1% Triton X-100, 50 mM HEPES, pH7.4, 150 mM NaCl, 1.5 mM MgCl_2_, 1 mM EGTA, 100 mM NaF, 10 mM Na pyrophosphate, 1 mM Na_3_VO_4_, 10% glycerol) and containing freshly added protease and phosphatase inhibitors. Protein concentration was measured using micro-bicinchoninic acid assay (Pierce, Rockford, IL). The protein concentration of each lysate is then diluted to 1 mg/mL using lysis buffer and the appropriate volume of 4× Laemmli buffer (40% glycerol, 8% SDS, 0.25 M Tris-HCl pH6.8) containing freshly added 2-mercaptoethanol (1:10). The samples were then heated to 95 °C for 5 min before using immediately for SDS-PAGE or storing for future use at −80 °C. Total protein (10 µg) was resolved using SDS-PAGE, transferred to nitrocellulose membranes and blocked in TBS-T/5% milk for 1 h. Membranes were incubated overnight at 4 °C with either: a rabbit monoclonal BOK antibody (clone 1–5) diluted 1:250^22,24^, a rabbit polyclonal Calreticulin antibody diluted 1:1000 (Cell Signalling Technologies, Dublin, Ireland), a mouse monoclonal KDEL antibody diluted 1:500 (clone 10C3, Enzo Life Sciences, Exeter, UK), a rabbit polyclonal Cleaved Caspase-3 (Asp175) antibody 1:500 (Cell Signalling Technologies, Dublin, Ireland) and a mouse monoclonal β-actin antibody diluted 1:5000 (clone AC-74; Sigma, Dublin, Ireland). The blots were then washed in TBS-T and incubated in the appropriate horseradish peroxidase secondary antibody (Pierce) diluted 1:10,000 at room temperature for 1 h. The KDEL antibody used binds the amino acid sequence Lys-Asp-Glu-Leu (KDEL) present at the carboxy-terminus of GRP78 and GRP94, allowing for both GRP78 and GRP94 detection. Detection of protein bands was carried out using chemiluminescence (EMD Millipore, Billerica, MA, USA) on a LAS-3000 Imager (FUJIFILM UK Ltd. Systems, Bedford, UK). We used 12-bit images to ensure that saturation limits were not reached, allowing for suitable quantitative detection method. We have previously used these methods also for absolute quantification studies^37^. Image J was utilised as image analysis software to perform densitometric evaluations of the specific bands (i.e., BOK, GRP78, GRP94, Calreticulin and cleaved caspase-3) relative to control bands (β-actin) in each blot. The background signal was compensated for, and we thus derived the relative quantity of our proteins of interest to determine tumour to matched normal ratios for each patient.

### *Bok* mutation, methylation and gene expression analysis on publicly available data sets

Two publicly available data sets were used to analyse *Bok* gene methylation status and gene expression level. We accessed TCGA preprocessed data (28/01/2016) through Firebrowse (firebrowse.org, Broad Institute TCGA Genome Data Analysis Center (2016): Analysis Overview for Colon Adenocarcinoma (Primary solid tumor cohort)—28 January 2016. Broad Institute of MIT and Harvard. doi:10.7908/C1F76BX1) with level 3 Methylation status plus patient follow-ups of COAD patients to analyse *Bok* gene methylation. The status of 14 methylation sites of the *Bok* gene were compared in matched normal and tumour tissues (*n* = 38) after which survival analysis was carried out. We also downloaded the NCBI Gene Expression Omnibus (GEO) GSE39582 (22/05/2013) data set^38^ that contains mRNA expression and patient follow-ups of the CIT CRC cohort. DFS and OS survival of patients (*n* = 556) were compared against high vs low levels of *Bok* mRNA.

### Statistical analysis

For the TCGA COAD and CIT cohorts, significance of *Bok* methylation sites differences between matched normal and tumour tissues was determined using Student’s *t*-test followed by Benjamini–Hochberg multiple correction. We used Kaplan–Meier log-rank tests to compare differences between survival curves. All of TCGA data analyses were performed within R (V.3.3.0, The R Foundation) including the survival analysis (using the ‘survival’ package V.2.39–4).

All results from the patient samples were analysed using the GraphPad InStat software. The expression of individual proteins in tumour and matched normal samples was compared with two-sided Wilcoxon signed-rank test for related variables.

Potential outliers were investigated using a structured unbiased approach. First, we evaluate whether measurements were outliers not only in the normal measurements but also in the context of tumour vs normal measurements. We then computed the difference between the expression of BOK in tumour and normal tissue and considered the points for potential outlier if (1) either the difference in expression between tumour and normal resulted in greater than *value* 75th + 1.5 × (*value* 75th − *value* 25th) or (2) lesser than *value* 25th − 1.5 × (*value* 75th − *value* 25th). The expression of the tumour to normal ratios was calculated using Mann–Whitney *U* test for independent samples. Results were considered significant when the *p*-value was <0.05.
